# Supplementary material for: Cultivated St. John’s Wort Flower Heads Accumulate Tocotrienols over Tocopherols, Regardless of the Year of the Plant
Source: Plants (Basel). 2025 Mar 9;14(6):852. doi: 10.3390/plants14060852 (PMC11945684; doi:10.3390/plants14060852)
Supplement: Supplementary file 1 [file plants-14-00852-s001.zip › plants-3506306-supplementary.pdf]

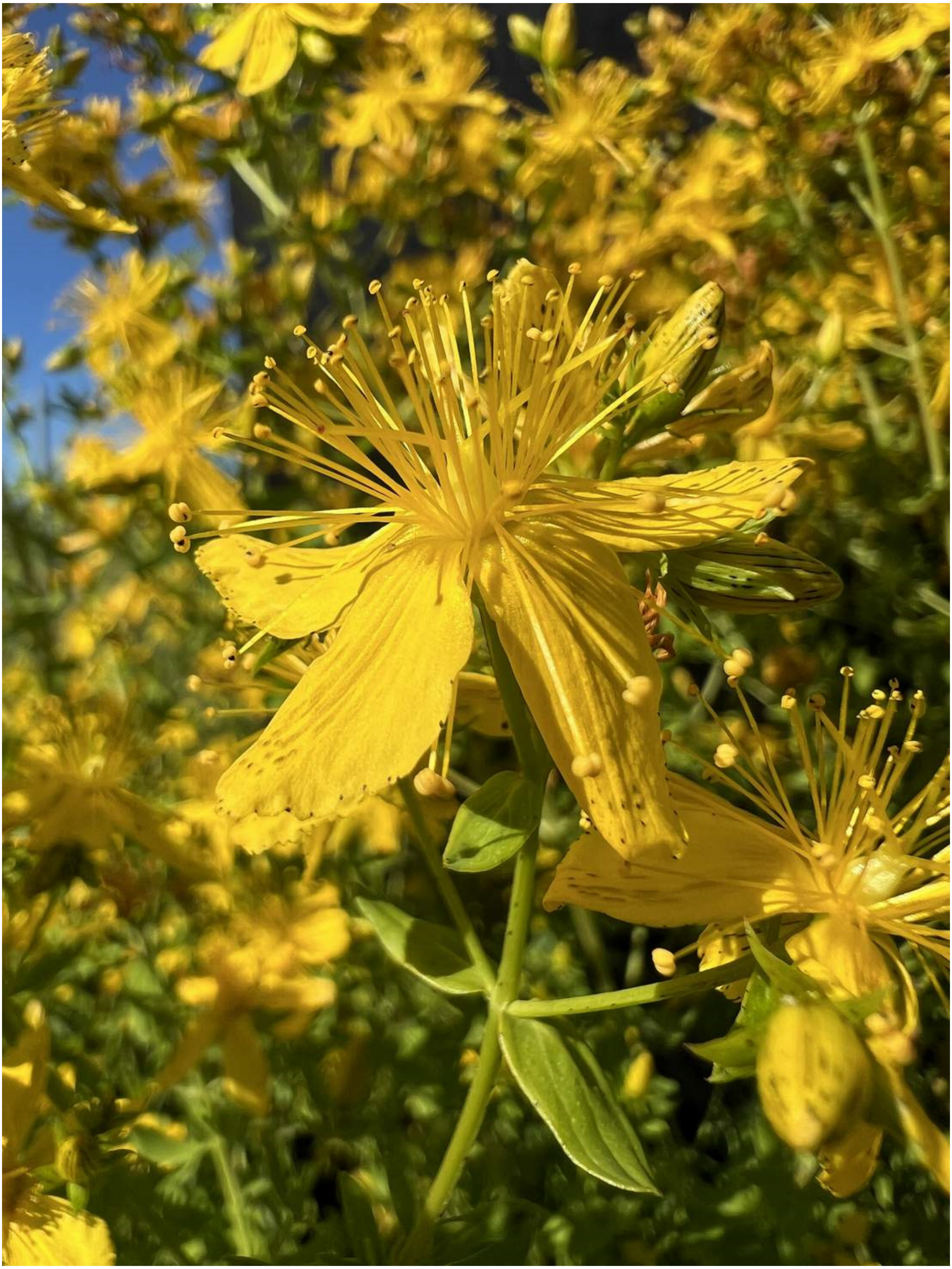

Figure S1. Cultivated St. John's wort (*Hypericum perforatum* L.).

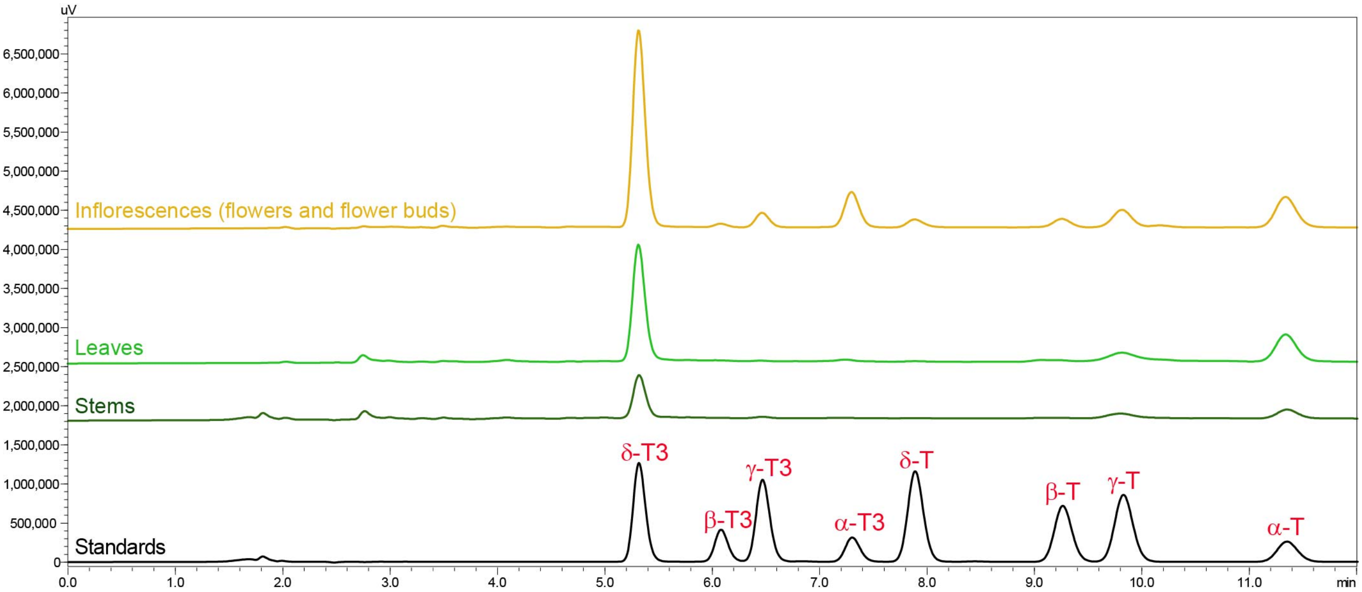

Figure S2. Chromatograms of the tocopherol (T) and tocotrienol (T3) homologues (α, β, γ, and δ) separation by RP-HPLC-FLD in cultivated *H. perforatum* stems, leaves, inflorescences (flower buds and flowers) and standards.
